# Computational simulation methodologies for mechanobiological modelling: a cell-centred approach to neointima development in stents

**DOI:** 10.1098/rsta.2010.0071

**Published:** 2010-06-28

**Authors:** C. J. Boyle, A. B. Lennon, M. Early, D. J. Kelly, C. Lally, P. J. Prendergast

**Affiliations:** 1 Trinity Centre for Bioengineering, School of Engineering, Trinity College Dublin, Dublin, Republic of Ireland; 2 Department of Mechanical and Manufacturing Engineering, Dublin City University, Dublin, Republic of Ireland

**Keywords:** cell-centred, lattice-based, restenosis, biological simulation, multi-scale model, cellular automaton model

## Abstract

The design of medical devices could be very much improved if robust tools were available for computational simulation of tissue response to the presence of the implant. Such tools require algorithms to simulate the response of tissues to mechanical and chemical stimuli. Available methodologies include those based on the principle of mechanical homeostasis, those which use continuum models to simulate biological constituents, and the cell-centred approach, which models cells as autonomous agents. In the latter approach, cell behaviour is governed by rules based on the state of the local environment around the cell; and informed by experiment. Tissue growth and differentiation requires simulating many of these cells together. In this paper, the methodology and applications of cell-centred techniques—with particular application to mechanobiology—are reviewed, and a cell-centred model of tissue formation in the lumen of an artery in response to the deployment of a stent is presented. The method is capable of capturing some of the most important aspects of restenosis, including nonlinear lesion growth with time. The approach taken in this paper provides a framework for simulating restenosis; the next step will be to couple it with more patient-specific geometries and quantitative parameter data.

## 1. Introduction

The potential of computational models for understanding biological systems is increasingly recognized, and their use in biomedical device design is increasing. Gait analysis, modelling of hard and soft tissues, fluid dynamics, etc. have enabled characterization of the mechanical function and dysfunction of the human body across many length scales, as can be seen, for example, from the progress made in the virtual physiological human (VPH) initiative (http://www.vph-noe.eu/vph-initiative). The VPH initiative has successfully elaborated on the functional complexity of many biomechanical systems, for example the cardiac system (Kohl & Noble 2009). One complexity that is central to biomechanical systems is their ability to alter their structure (through growth and differentiation)—there is much scope to address this type of complexity within the VPH project. Many of the most common health issues in the skeletal and cardiovascular systems stem from the property of adaptation over medium (weeks) to long (years) time scales, such as osteoporosis, cardiovascular diseases, developmental abnormalities, wound/fracture healing, ageing and biomedical device failure. The biomechanical system can adapt by changing its shape, such as in bone remodelling, hypertension-induced arterial narrowing and tumour growth, or by altering the mechanical properties of the constituent materials, e.g. tissue differentiation and atherosclerosis. Mechanics plays a key role in regulating this adaptation: mechanobiological models are required to capture the long-term behaviour of the system, and if such models can be successfully corroborated, it opens one way to improve the design of medical devices.

Tissue adaptation can be represented as a system striving to achieve a homeostatic mechanical state by altering its geometry (growth/resorption) or material properties (stiffness, porosity, etc.). This concept has been successfully applied to bone remodelling (Cowin & Hegedus 1976; Huiskes *et al*. 1987; Prendergast & Taylor 1994) and arterial remodelling (Rachev *et al*. 2000; Kuhl *et al*. 2003; Alastrue *et al*. 2008). However, this concept of reverting to a supposed homeostatic equilibrium hides a complexity because adaptation processes are indirectly related to mechanics, through complex systems of cells, tissue and chemical signals. In skeletal tissue differentiation, for example, the differentiation of mesenchymal stem cells is mechanoregulated, producing a complex link between mechanics and tissue adaptation. This complexity led to the explicit inclusion of cell activity in mechanobiology, where cell behaviours such as proliferation, differentiation and migration were modelled with differential equations (Lacroix *et al*. 2002; Bailon-Plaza & van der Meulen 2003; Lally & Prendergast 2004), or where cell activity was indirectly modelled through evolution equations (Zohdi *et al*. 2004; Ruimerman *et al*. 2005). Continuum assumptions break down at the level of the cell, which are typically 10–100 µm in diameter. In arteries, which are of the order of 500 µm thick, this means the organ may only be 5–50 cells thick.

The hierarchical structure of biomechanical systems permits modelling at various length scales; from the tissue level, through the cell level to the molecular level. Formulating the model at the tissue level requires a phenomenological link between stress and adaptation which may be a highly complex relationship. Indeed, there may be no adequate equations available to model this relationship. At this level, the system is slow to react to the mechanical environment; it reacts over months, whereas at the other extreme, mechanics affect the system very rapidly, by opening ion channels, or altering the morphology of the cells. Models at this level would be the most complex, as all intercellular and extracellular processes would be important, and modelling them would quickly become unmanageable. At the level of the cell, we can postulate that mechanics affects the behaviour of cells, i.e. by regulating differentiation, apoptosis, proliferation, etc. One major benefit of this approach is that much of experimental biology is focused on this level (Prendergast *et al*. 2009; Checa *et al*. 2010). The key to effective modelling of tissue adaptation is a multi-scale approach (Evans *et al*. 2008)—where processes at the continuum/tissue level (for example stress/strain), and at the intercellular level (perhaps gene transcription) can inform processes at the cell level (differentiation, proliferation, etc.).

## 2. Neointima formation

Muscular arteries are tubular vessels of smooth muscle surrounded by an endothelium on the inner surface. Atherosclerosis is an asymmetric focal thickening of the artery wall, which can lead to occlusion, depriving downstream tissues of oxygen, which can lead to myocardial infarction or stroke. Stents have become one of the most popular treatment techniques; however, different stents produce different amounts of restenosis (Hoffmann *et al*. 2001, 2002; Morton *et al*. 2004). Restenosis is due to tissue in-growth into the lumen as a result of injury to the vessel wall imparted by the expanded stent. The smooth muscle cells (SMCs) which populate the artery wall have complex behaviours, but a simplified description (see Thyberg *et al*. 1990) is the following.

— SMCs can exist in two distinct phenotypes: contractile (cSMC) and synthetic (sSMC). The cSMCs exist in the uninjured tissue, and remain quiescent (do not proliferate or synthesize matrix) unless the extracellular components are disrupted.
— If the extracellular matrix (ECM) in injured regions is disrupted/degraded, cSMCs modulate their phenotype and become synthetic—producing ECM and responding to growth factors by proliferating.

Neointima formation in response to injury can be simplified into distinct, overlapping phases (Drasdo *et al*. 1995; Evans *et al*. 2008). An exudative phase occurs when macrophages and other inflammatory cells aggregate around and within the injury site from the blood. They initiate a resorptive phase where debris, tissue and dead cells are decomposed by inflammatory cells. These cells, together with the dead SMCs and endothelial cells (ECs) produce growth stimuli, which initiate the proliferation phase where SMCs proliferate and infiltrate the injured region, depositing granulation tissue. The repair phase then consists of deposition of collagen-based tissue in place of the granulation tissue. The endothelium is influential in providing a mechano-sensitive barrier between the smooth muscle and the blood, and can also actively signal SMCs. This layer is damaged (often removed completely) during stent deployment and its growth-inhibitory effects are only restored when the layer re-heals.

The above description is a simplified, mechanistic description of the process of neointima formation in the artery. In this paper, we explore the use of the cell-centred approach to develop a predictive model for restenosis. We apply the approach to restenosis development within an artery after implantation of a stent, and explore the impact of EC proliferation rate on the development of restenosis.

## 3. Theoretical framework of cell-centred methodologies

Cell-centred approaches to adaptation aim to reproduce the complex dynamics of the biological system through modelling the behaviour of individual cells. They require a representation of cells (spatial and behavioural), and may include environmental variables (biochemical factors, ECM molecules, mechanics). The system is incremented by applying behavioural rules to cells based on the local environment.

In reality, cells are discrete entities and have a complex and dynamic structure. When producing discrete cell-based models, some coarsening/simplification of the cell structure is necessary. Lattice-based methods discretize the spatial domain into a regular orthogonal grid of points, in which case the cells occupy either individual unique nodes (like a cellular automaton; Peréz & Prendergast 2007), or occupy several adjacent nodes, as in the cellular Potts model (CPM; Graner & Glazier 1992; Glazier & Graner 1993; Izaguirre *et al*. 2004; Cickovski *et al*. 2005; van Oers *et al*. 2008*a*,*b*), explained below. In non-lattice-based approaches (Drasdo *et al*. 1995; Walker *et al*. 2003), the cells have a defined location in continuous space, and a volume associated with this. In this approach, the neighbourhood must be searched using spatial search algorithms, such as octrees, which means, for large numbers of cells, the computation time of non-lattice techniques can grow massively. The lattice approach offers a less computationally expensive method, as it provides a grid within which the model variables are defined, meaning that the sampling of the environment is reduced to sampling adjacent lattice sites. Non-lattice techniques allow more complexities, like growth forces (Walker *et al*. 2003), cell deformations and growth (Drasdo *et al*. 1995), to be dealt with. The decision over which of these representations to use will be based on the length scale and size of the problem.

Components, such as diffusible chemicals, ECM, etc. and biophysical stimuli, such as strain and fluid flow, can be represented as spatial fields. Field variables can be implemented as having continuously varying values at discrete points and can be defined at the points of the cell lattice (in structured grids), or interpolated from a separate grid, such as a finite element mesh (Byrne 2008). Mapping from coarser grids in this way enables multi-scale modelling of biophysical stimuli that may be more suited to a coarser representation while retaining sufficient biological detail to model processes of interest at the cellular level.

Cell types relevant to most mechanobiological models have a limited set of behaviours: proliferation, migration, differentiation, absorption/expression of chemicals and apoptosis. Cells move through tissue by crawling. Cell migration can be represented as a random walk process. This can include a random direction, a random distance, and can be isotropic or chemo/haptotactic. Random walk within a lattice is implemented by allowing the discrete cells to jump to available neighbouring positions (selected at random—or biased along the gradient of a stimulus). The CPM, developed by Glazier and Graner (Graner & Glazier 1992; Glazier & Graner 1993) from a lattice-based model for grain-coarsening in metals, represents cell movement as a fluctuation of the cell boundary where lattice points are added/removed from the cell at random, provided the change meets requirements for cell volume and surface area. The cell cycle itself can be implemented, where cells pass through phases based on time and external cues. Non-lattice-based methods can use an overlapping force technique to represent the multi-body mechanics of cell growth, allowing cells to overlap and exert forces on their neighbours. This may be important to study residual stress formation during tissue growth. These behaviours are then implemented as explicit updating procedures.

In lattice-based approaches, every lattice point can be operated upon sequentially or randomly; however, when the lattice is dense and sparsely populated, this can be inefficient. Instead, a list of cells in the lattice can be computed at the beginning of a simulation, and the simulation operates on each cell in the list. Non-lattice-based approaches also operate on each cell in the model; however, spatial searching of an unstructured space must be used (see above).

The cell-centred technique is extremely versatile, and has been used to simulate a wide variety of biological systems. Developmental processes, cell sorting and tumour growth have been simulated using the CPM (see the review by Merks & Glazier 2005). Dermal wound healing has been simulated in three dimensions and wound healing assays have been simulated using agent-based techniques (Walker *et al*. 2003; Mi *et al*. 2007; Simpson *et al*. 2007). The lattice-based approach has been used in a wide variety of mechanobiological models. Peréz & Prendergast (2007) were the first to use the random walk approach for cell proliferation and migration in a simulation of tissue differentiation. In this model, the migration and proliferation of mesenchymal stem cells were simulated in a lattice. Finite element analysis was performed at regular intervals to determine the mechanical stimuli of fluid flow and shear stress, which regulated the differentiation of these cells into osteoblasts, chondrocytes or fibroblasts, following the theory proposed by Prendergast *et al*. (1997). These differentiated cells produced their characteristic tissues, which then altered the mechanical properties of the tissue, and hence the stimulus. In this way, the time-course of tissue differentiation could be captured in a simulation. This was later applied to idealized bone scaffolds (Byrne *et al*. 2007), fracture healing (Byrne 2008), tissue differentiation in a bone chamber (Khayyeri *et al*. 2009), and tissue differentiation with angiogenesis (Checa & Prendergast 2009). In that study, angiogenesis was included by modelling the capillaries as sprouts of adjacent ECs. These then influenced tissue differentiation, as bone formation was assumed to only occur within a specified distance of an oxygen source (capillary). Van Oers *et al*. (2008*a*,*b*) have used the CPM to simulate osteon formation, and bone remodelling through the explicit modelling of osteoclast behaviour. In this model, osteoclasts are assumed to attach to bone sites where a mechanical stimulus signal (sensed by osteocytes in the bone) is low. Osteoblasts produced bone at the newly exposed surface. Osteonal remodelling then occurred through the sequential resorption and deposition of bone.

Preliminary results from the COAST project (www.complex-automata.org) have shown the possible power of a multi-scale, multi-physics model of restenosis development (Caiazzo *et al*. 2009). Blood flow, structural stress and diffusion from a stent were simulated and combined with an agent-based model of SMC behaviour in two dimensions. This model assumed that SMC proliferation was governed by local fluid flow, structural stress and drug concentration. A tumour-like growth pattern was found, which was inhibited by the presence of drugs.

As the cell-centred methodology matures, it will find more applications, as it has the ability to incorporate a maximal amount of experimental information. In the next section, a cell-centred approach is applied to restenosis development within an artery due to stenting.

## 4. Application to neointima development

**Figure 1.**
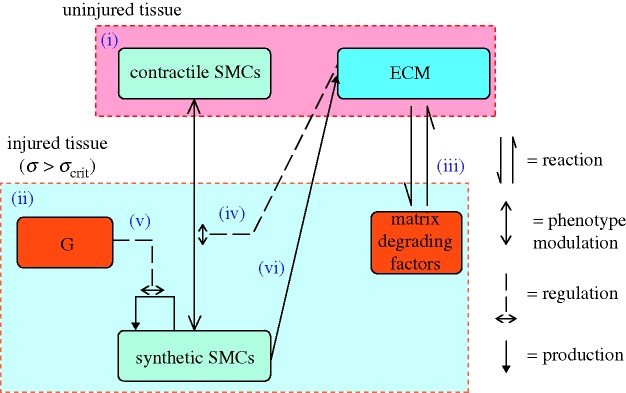
A system diagram of the components (in boxes) and processes (arrows) involved in the model of smooth muscle cell behaviour. (i) The uninjured artery wall is quiescent and contains contractile smooth muscle cells (cSMCs) and extracellular matrix (ECM). Injury induces cell death and tissue damage/rupture. (ii) In response, inflammatory cells infiltrate the injured region and produce matrix degrading factors and growth factors. (iii) Matrix degradation of ECM induces (iv) SMCs to modulate their phenotype from contractile to synthetic (sSMC). These sSMCs proliferate in response to growth factors (G) (v) and express ECM (vi). SMCs can resort back to the contractile phenotype if the ECM is fully restored.

**Figure 2.**
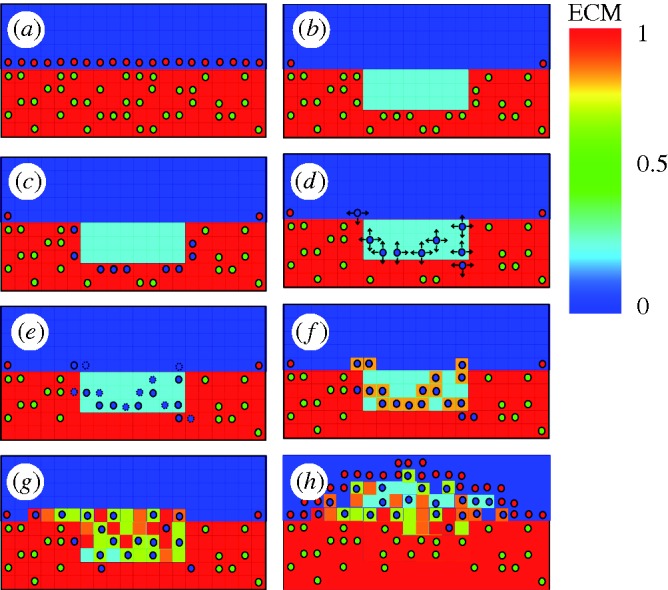
Schematic diagrams of the rules governing cell activity in the lattice model. (*a*) SMCs surrounded by ECM and with an intact endothelium express a contractile phenotype, (*b*) ECM is reduced in injured areas, simulating the degradation of ECM by MDFs, and SMCs are removed (*c*). cSMCs neighbouring the reduced ECM region modulate to the synthetic phenotype, (*d*) sSMCs migrate by random walk (arrows indicate possible movements) and (*e*) proliferate (dashed lines indicate daughter cells), (*f*) ECM is produced by cells as they move, and (*g*) over time a lesion forms until (*h*) the endothelium has healed. A video of this process in action is included as the electronic supplementary material, video S1 (green circles, cSMC; blue circles, sSMC; red circles, EC).

A cell-centred model of an idealized human coronary artery was implemented using a lattice-based approach. Three cell types (cSMCs, sSMCs and ECs), and three extracellular components (ECM, matrix degrading factors (MDF), and growth stimuli (G)) were modelled. The SMC phenotype was governed by the local ECM concentration: it was contractile if the ECM was present and not degraded (ECM = 1) and the local concentration of cells was below a critical value *c*_SMC,crit_, and synthetic otherwise. This ensured that a region would return to quiescence when the ratio between cells and ECM was appropriate. Differentiation was assumed to be reversible. sSMCs proliferated if the local growth stimulus was above a critical level (*G*_crit_), and occurred at a constant rate (*p*_SMC_). A successful proliferation reduced the local growth stimulus by *G*_crit_. Migration of sSMCs occurred in random directions at a constant rate (*v*_SMC_) through lattice points which either had or were adjacent to points with ECM present. sSMCs produced ECM at a constant rate (*c*_ECM_) at their lattice position. In lattice points containing both ECM and MDF, both of these components were reduced at a constant rate of degradation, *c*_deg_. ECs were only allowed to occupy lattice points which fulfilled the following conditions: they did not contain ECM, and they were adjacent to a lattice point with ECM, i.e. ECs could only proliferate along the lumen surface, and occurred at a constant proliferation rate, *p*_EC_. A system diagram of the simplified system implemented is given in figure 1. The lattice was updated according to these rules over time, as shown schematically in figure 2. At each time increment, all cells were operated upon based on their phenotype. Random walk migration and proliferation were performed on each active cell a number of times per increment based on the migration and proliferation rates, respectively. Differentiation of SMCs and ECM production were performed every increment, with the increment corresponding to one day. Depending on the particular initial conditions and parameters of the model, this simulation can progress until equilibrium is reached, i.e. when the endothelium is completely healed, or until the lumen is entirely occluded. SMCs cannot occupy a lattice point containing another cell, so a position occupied by an EC represents an immovable and impenetrable barrier to SMCs.

**Figure 3.**
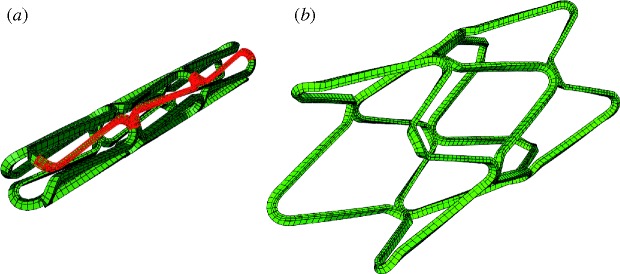
Finite element mesh of the unexpanded stent (*a*) and after expansion in a cylindrical artery (*b*). For computation, the stent and artery were reduced down to a 1/8 model in the circumferential direction, the modelled portion of the geometry can be seen in red.

Finite element analysis was performed and coupled with an injury threshold to quantify injury in the artery wall due to stenting. The artery was modelled as a hollow cylinder with a length of 14 mm and inner and outer diameters of 2.5 and 3.75 mm, respectively. Following Gervaso *et al*. (2008), the material properties of the artery were represented as a third-order Mooney–Rivlin hyperelastic model based on circumferential, uniaxial tests carried out on non-diseased media from human coronary arteries with intimal thickening by Holzapfel *et al*. (2005). A four-crowned, corrugated-ring, stainless-steel stent was modelled with similar designs to those used by Garasic *et al*. (2000); the finite element (FE) mesh of the expanded and unexpanded stent is shown in figure 3. The stent was modelled as a bi-linear elasto-plastic material with the properties of stainless steel (Early *et al*. 2009). Physiological conditions in the artery were simulated by applying an axial pre-stretch of 1.2 and a blood pressure of 13.3 kPa prior to stent expansion. A radial displacement was applied to all nodes of a cylinder within the stent, expanding it from an initial diameter of 1.15 to 3.3 mm. This achieved an expansion ratio of 1.2. The cylinder was retracted allowing recoil of the stents. To reduce the simulation times, symmetry was used to reduce to a 1/8th model circumferentially (figure 3). Appropriate boundary conditions were applied along the planes of symmetry. The simulations were run using ABAQUS (SIMULIA) to determine the stresses in the artery following stent expansion.

In order to map stress from the FE mesh onto the lattice, the lattice bounds were initialized to encompass the entire FE mesh. For each element in the FE mesh, the lattice points within that element were found following the method of Byrne (2008). Briefly, a bounding box was formed around each element, with each lattice point within this box tested to check if it fell within all six faces of the element. These points were initialized as artery lattice points by assigning the stress value of the element and by initializing them with ECM = 1. cSMCs were randomly distributed within a certain percentage (*c*_SMC_) of the arterial lattice points. Injury was assumed to occur above a critical threshold of minimum principal stress. At lattice points with a stress above this value, cells were removed and initial conditions for G and MDF were introduced (table 1). These represent an acute source of injurious factors, and encompass production from SMCs, EC, platelets and inflammatory cells. ECs were initialized to cover the lumen surface proximal and distal to the stent ends, simulating total denudation within the stented region, as has been found for balloon-expanded stents (Harnek *et al*. 1999). The model assumes endothelium heals through proliferation of healthy ECs in neighbouring regions to the stent. The simulation was then run for an equivalent of 320 days post-stenting. The neointimal area at an axial position within the stent was calculated as the area of lattice points containing ECM minus the initial cross-sectional area of the artery. The volume of the lesion was calculated as the sum of these areas over the length of the artery. Cell numbers were counted, and the cumulative population doubling of the cells per day was calculated (measuring overall proliferation rate) as log_2_(*N*/*N*_0_), where *N* is the current number of cells and *N*_0_ is the previous day's cell number.

**Table 1.** Parameter estimates used in the baseline simulation.

parameter	symbol (units)	value
lattice spacing	*dl* (mm)	0.01825^a^
stress threshold	σ_crit_ (kPa)	35 (compressive)^b^
initial smooth muscle cell concentration	*c*_SMC,init_ (cells mm^−3^)	3.16 × 10^4 c^
smooth muscle cell migration speed	*v*_SMC_ (mm d^−1^)	0.24^d^
smooth muscle cell proliferation rate	*p*_SMC_ (mitoses d^−1^)	0.24^a^
endothelial proliferation rate	*p*_EC_ (mitoses d^−1^)	2^e^
initial matrix degrading factor	MDF_init_ (dimensionless)	1^e^
extracellular matrix degradation rate	*c*_deg_ (dimensionless)	0.05^e^
extracellular matrix production rate	(dimensionless)	0.20^e^
initial growth factor	G (dimensionless)	3^a^

^a^Schwartz *et al*. (1996).

^b^Linder-Ganz *et al*. (2006).

^c^Tracy (1997).

^d^DiMilla *et al*. (1993).

^e^Chosen to agree with time-course of restenosis studies/reviews (Edelman & Rogers 1998; Orford *et al*. 2000; Bennett & O'Sullivan 2001; Welt & Rogers 2002).

The model parameters were selected based on experimental values, calibrated against experiments or selected based on the descriptions of the time-course of neointima development. DiMilla *et al*. (1993) measured the migration speed of individual human SMCs on various substrates *in vitro* and found the speed to be of the order of 10–20 µm h^−1^. The proliferation dynamics of SMCs in the artery are controlled by the proliferation rate and amount of initial growth stimulus. These parameters were selected to produce a similar proliferation profile to human SMCs (Schwartz *et al*. 1996). Matrix degradation was assumed to completely resorb damaged tissue (in the absence of matrix producing cells) after 20 days. The endothelial healing rate was selected to achieve re-endothelialization of an 8 mm denuded artery after 180 days (giving *p*_EC_ =2). Due to the large variation in this parameter, it was varied over a wide range (proliferation attempt rate of 0–4 per cell per day). The lattice density represents the maximum cell density possible. This was set to 1.64 × 10^5^ cells per mm^3^, based on human cell concentrations in neointima (Schwartz *et al*. 1996). The initial concentration of SMCs was based on cell concentrations in human artery tissue (Tracy 1997), and set to 3.16 × 10^4^ cells per mm^3^. Experiments on skeletal muscle have shown that the threshold of compressive stress for skeletal muscle cell viability is between 35 and 70 kPa (Linder-Ganz *et al*. 2006), and the lower limit of 35 kPa was used as the injury threshold (σ_crit_) for arterial smooth muscle in the simulations presented here. Parameters are summarized in table 1.

## 5. Results

**Figure 4.**
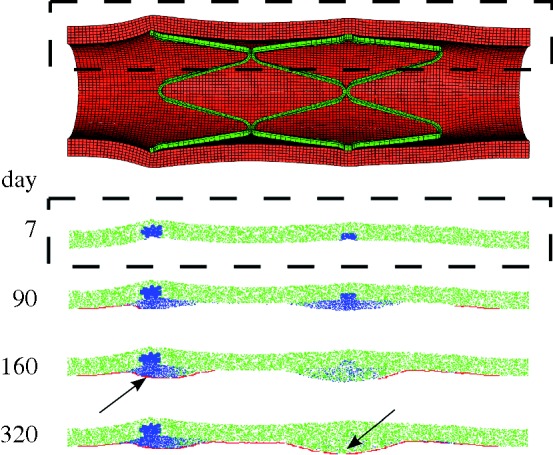
The expanded stent within the artery (top). The region bound by the dashed box was monitored for cell states over the simulation. At day 7, sSMCs are active in the injured region. The lesion progresses until the endothelium covers the tissue (arrow, day 160) or until the SMCs resort to their contractile phenotype (arrow, day 320). Simulation shown is for endothelial proliferation rate, *p*_EC_ = 2 (green, cSMC; blue, sSMC; red, EC).

**Figure 5.**
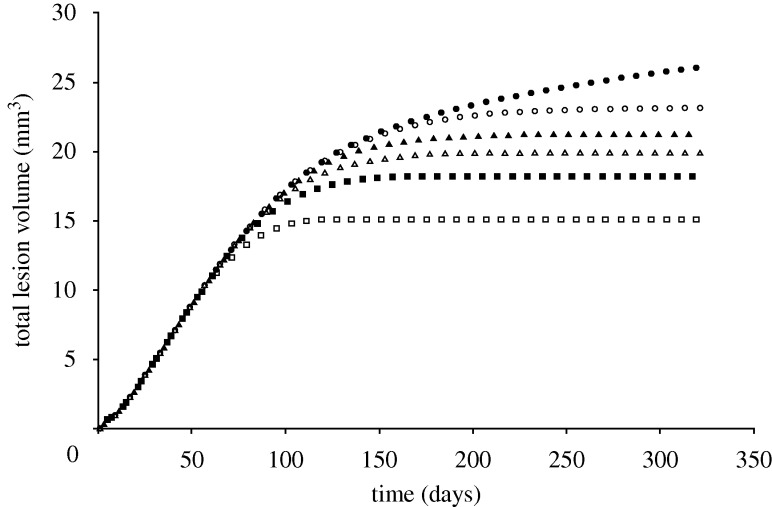
As the endothelial cell proliferation rate increases, the volume of neointima produced reduces (*p*_EC_ = 0 (filled circle), *p*_EC_ = 1 (open circle), *p*_EC_ = 1.5 (filled triangle), *p*_EC_ = 2 (open triangle), *p*_EC_ = 2.7 (filled square), *p*_EC_ = 4 (open square)).

**Figure 6.**
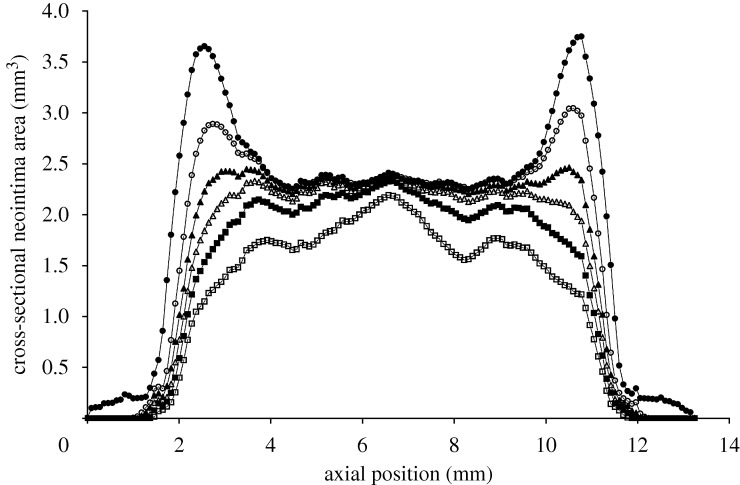
As endothelial proliferation rate decreases, maximum neointima at the stent ends at 320 days increases (*p*_EC_ = 0 (filled circle), *p*_EC_ = 1 (open circle), *p*_EC_ = 1.5 (filled triangle), *p*_EC_ = 2 (open triangle), *p*_EC_ = 2.7 (filled square), *p*_EC_ = 4 (open square)).

**Figure 7.**
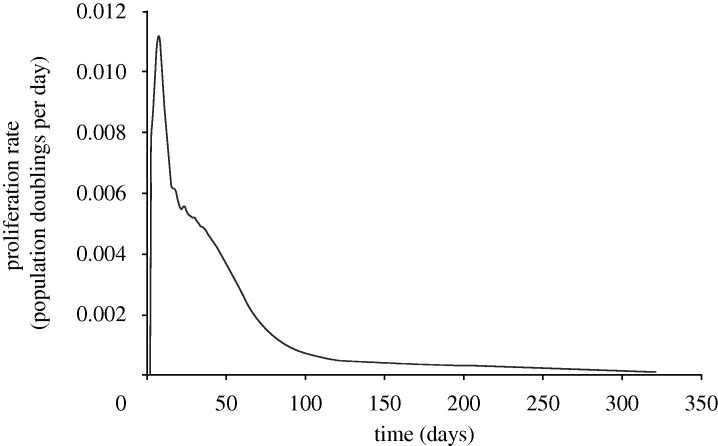
Proliferation rate, measured as cumulative population doublings of neointimal SMCs per day in the artery over time, for *p*_EC_ = 2. Endothelial healing rate was found to have minimal effects on the proliferation profile of the artery (results for other values of *p*_EC_ not shown for clarity).

Injury was predicted under stent struts, with the highest injured volumes at the stent ends and at stent cell junctions. ECM degradation in these regions induced phenotype modulation to sSMCs in surrounding cSMCs. These sSMCs migrated into the injured regions and proliferated to fill up the injured space (figure 4, day 7) eventually leading to lesion growth (figure 4, days 90 and 160). Lumen narrowing was arrested in two ways. In regions where the injured volume was small, such as in the centre of the stent, sSMCs modulated back to the contractile phenotype. In regions with a large injured volume, such as at the ends of the stent, the amount of sSMCs was greater, and lumen narrowing did not stop until the healing endothelium covered it (figure 4, day 320). In this region, SMCs did not return to their contractile phenotype within the duration of the simulation. The lesion grew rapidly in the first 90 days for all simulations (figure 5). For high *p*_EC_ rates, the lesion growth then slowed to a stop when the endothelium healed (approx. 180 days), whereas for low *p*_EC_ rates the growth rate simply slowed (figure 5). The final distribution of the lesion was also highly dependent on endothelial proliferation rate, with low healing rates associated with focal thickening at the ends of the stent and high healing rates producing maximal lesion size at the centre of the stent (figure 6). The proliferation rate over time of the artery was largely unaffected by changes in *p*_EC_; all simulations produced a peak in proliferation early on, followed by a gradual reduction in proliferative activity (figure 7).

## 6. Discussion

Many of the structural changes that biological systems undergo are complex, and are a result of many biological components interacting. It has been argued that producing mathematical laws governing such a complex system is difficult: ‘to do so would be like expecting to discern the fundamental laws of electromagnetism in the output of a personal computer’ (Lander 2004), indeed, no such laws may exist. An alternative is to break up the system, until the components begin to behave according to simple models. The extreme of this path is to reduce down to the molecular level, where reaction–advection–diffusion equations govern. Even if this were possible for any relevant model size, it may not be useful, as the model system would be just as complicated as the reality (Merks & Glazier 2005). The cell is a natural level of abstraction, as they are discrete entities and their behaviour can be reduced to a simple set of rules. They process inputs such as extracellular environment, mechanical stimuli and chemical stimuli, and regulate their behaviour accordingly to migrate, proliferate, secrete extracellular components, produce force, apoptose, etc. The aim of the modeller is then to develop a sufficiently accurate model of individual cell behaviour that, when combined with many cells in a simulation, produces a robust, and accurate, tissue-level model.

The example described here has some limitations. The geometry of the problem was simplified to a cylinder, whereas in patients, a significant stenosis with heterogeneous properties would affect the calculations of stress (and therefore injury) in the artery (Kiousis *et al*. 2007; Gijsen *et al*. 2008). Compressive stress was chosen as the stimulus causing tissue injury; however other variables such as strain, tensile stress or strain energy density may better predict injury/damage/cell death. More information is needed on the relationship between mechanics and initial injury. The inflammatory response was modelled only as an initial condition of MDFs and growth factors. In reality this is a complex response, which occurs as monocytes, macrophages and platelets invade the injured zones from the lumen and act locally to remove debris and stimulate SMCs. This process may need to be modelled more explicitly by including macrophages. The parameters for the behaviours of the cells in the system have been taken from *in vitro* and *in vivo* data from various species. These parameters have an effect on the outcome of the simulations, and will need to be explored experimentally. The endothelium healing rate was found to have a large effect on the amount of restenosis. Endothelial proliferation rate varies considerably, and the healing rate is dependent on the injury of the underlying tissue (Kipshidze *et al*. 2004), underlying substrate strain (Moretti *et al*. 2004) and fluid flow-induced shear stress (Zarins *et al*. 1987; White *et al*. 2001; Resnick *et al*. 2003; Wentzel *et al*. 2003). The endothelium model implemented here represented a healed endothelium as a rigid, physical barrier to growth. In reality, the endothelium inhibits growth by acting as a barrier to growth stimuli, and underlying tissue can continue to grow. However, even with such simplifications, the simulation could capture important characteristics of lesion growth found *in vivo* in the following ways: lesion development was found to be nonlinear in time; unevenly distributed within the stent; and the endothelial healing rate was found to have a large effect on the progression of restenosis. In the future, a method for allowing sub-endothelial growth could be implemented by allowing displacement of ECs by smooth muscle.

This is very much a physiomic modelling approach, allowing the synthesis of a diverse range of information. Components may be studied in relative isolation, allowing the implementation of *in vitro* work, and the cell models should be generic enough to apply to a range of applications. The cell models underlying the cell-centred techniques must be based on sound rules, extracted from experimental observation, as the model behaviour is a product of these rules.

One current limitation of the cell-centred technique when applied to restenosis is the paucity of *quantitative* information on the dynamics of the system. However, if the loop between experimentation and theory is to be followed, more data are needed on key behaviours of cells within their natural environments rather than *in vitro*. This would require a benchmark experiment which is controlled to the highest degree possible; such experiments have informed skeletal tissue differentiation models (Khayyeri *et al*. 2009). Until such data are available, the simulation methodology is useful in exploring the possible behaviours of the system (Peck 2004).

A key benefit of including more biological processes in a model of tissue adaptation is the ability to make predictions about what happens when these processes are altered by the presence of an implant. As biomedical devices become more biologically active (such as drug-eluting prostheses or tissue-engineered implants), predictions from traditional models become more difficult. Also, biological parameters can be highly variable between patients, meaning a pure mechanical analysis may not accurately predict a device's behaviour in a population, as each patient's reaction to an identical stimulus will be different. Including biology allows the effect of this source of variation on the system to be studied. The COAST project aims to include growth-inhibiting drug diffusion in a multi-scale, multi-science model of restenosis development. The preliminary work assumes SMC proliferation is induced in response to high structural stress, or low wall shear stress, providing contact inhibition and drug concentration are acceptable. In contrast, the model described here includes explicitly the ECM (which constitutes a large proportion of restenotic tissue), growth factors (which control SMC proliferation, are diffusible, locally expressed and often short lived) and endothelium (which is influential in regulating smooth muscle tissue activity). The inclusion of these complexities provides a cell-centred model whose rules are based on the known biological mechanisms of restenotic tissue development. The regulatory effects of mechanical stimuli can then be implemented through a known/postulated link, such as wall shear stress regulating the production of growth factors at the lumen surface, or tissue stress inducing cell death and subsequent inflammation.

The gold standard for preclinical testing is currently still animals, particularly in predicting the medium to long-term response to an implant. However, their limitations in many key applications have been increasing. Their biological and mechanical systems are not accurate in predicting performances in many cases (see, for example, the review of animal models on drug-eluting stents by Schwartz *et al*. 2004). Provided that methodologies are adopted which can synthesize the independent islands of knowledge, computational models may well overtake animal models in the prediction of tissue adaptation.

## 7. Conclusions

Cell-centred methodologies have proven insightful and predictive in many diverse areas of biological system modelling, and represent a promising paradigm for including some of the temporal aspects of the VPH project.
